# Spider Mites Cause More Damage to Tomato in the Dark When Induced Defenses Are Lower

**DOI:** 10.1007/s10886-020-01195-1

**Published:** 2020-06-26

**Authors:** Jie Liu, Rachid Chafi, Saioa Legarrea, Juan M. Alba, Tomas Meijer, Steph B. J. Menken, Merijn R. Kant

**Affiliations:** 1 Section Molecular and Chemical Ecology, Department of Evolutionary and Population Biology, Institute for Biodiversity and Ecosystem Dynamics, University of Amsterdam, Amsterdam, Netherlands; 2 State Key Laboratory of Rice Biology & Ministry of Agriculture Key Lab of Molecular Biology of Crop Pathogens and Insects, Institute of Insect Sciences, Zhejiang University, 310058 Hangzhou, China

**Keywords:** Plant defense, Diurnal, Herbivore, Jasmonate, Salicylate, Effector

## Abstract

Plants have evolved robust mechanisms to cope with incidental variation (e.g. herbivory) and periodical variation (e.g. light/darkness during the day-night cycle) in their environment. It has been shown that a plant’s susceptibility to pathogens can vary during its day-night cycle. We demonstrated earlier that the spider mite *Tetranychus urticae* induces jasmonate- and salicylate-mediated defenses in tomato plants while the spider mite *T. evansi* suppresses these defenses probably by secreting salivary effector proteins. Here we compared induction/suppression of plant defenses; the expression of mite-effector genes and the amount of damage due to mite feeding during the day and during the night. *T. urticae* feeding upregulated the expression of jasmonate and salicylate marker-genes albeit significantly higher under light than under darkness. Some of these marker-genes were also upregulated by *T. evansi*-feeding albeit to much lower levels than by *T. urticae*-feeding. The expression of effector 28 was not affected by light or darkness in either mite species. However, the expression of effector 84 was considerably higher under light, especially for *T. evansi*. Finally, while *T. evansi* produced overall more feeding damage than *T. urticae* both mites produced consistently more damage during the dark phase than under light. Our results suggest that induced defenses are subject to diurnal variation possibly causing tomatoes to incur more damage due to mite-feeding during the dark phase. We speculate that mites, but especially *T. evansi*, may relax effector production during the dark phase because under these conditions the plant’s ability to upregulate defenses is reduced.

## Introduction

Plants have evolved sophisticated traits that enable them to handle the common variation in their environment. Some of this environmental variation is rather predictable, such as the daily light-dark cycle or the seasonal changes in temperature. However, environmental variation can also be less predictable, such as the emergence and disappearance of herbivores, predators and diseases. Therefore, adaptations to such variations often come down to a mixture of traits that are either being expressed constitutively or periodically, or that can occasionally be activated in response to a specific stimulus such as herbivory or light and can be inactivated again later (Angelmann and Johnsson, 1998; Atamian and Harmer 2016; Yakir et al. 2007). Plant traits that are specifically induced by herbivory are often related to damage repair and defenses. These can include reinforcement of mechanical barriers such as cell walls, the accumulation of toxic chemicals and feeding inhibitors and the attraction and/or arrestment of natural enemies of herbivores, which is often mediated via specific plant volatiles (Walling 2000). Inducible defenses are generally believed to be adaptive since it may reduce the costs relative to constitutive defenses (Rhoades and Cates 1976) while at the same time minimizing the risk of self-intoxication. The central regulators of inducible plant defenses are the phytohormones jasmonic acid (JA) and salicylic acid (SA). The former primarily orchestrates defenses against herbivores (Howe and Jander 2008) and necrotrophic pathogens (Glazebrook 2005), whereas the latter does so primarily against biotrophic pathogens and phloem-feeding herbivores (Kaloshian and Walling 2005). JA and SA signaling influence each other’s mode of action. This interaction is often - albeit not exclusively – antagonistic (Mur et al. 2006) and this may enable the plant to tailor distinct defense responses to distinct attackers (Thaler et al. 2012). How such specificity comes about is still poorly understood (Bonaventure et al. 2011) but it is generally believed that the combination of mechanical damage caused by feeding activities and secretion of (salivary) effector molecules can be perceived by the plant such that different types of attackers can be distinguished (Howe and Jander 2008; Hogenhout et al. 2009). Effector molecules have been in the center of attention especially in the field of phytopathology but also, in more recent years, in the field of plant-herbivore interactions. Many feeding-associated secreted substances, which are often, but not exclusively, proteins, were found to either elicit or to suppress inducible plant-defense responses (Hogenhout and Bos 2011). Elicitation and suppression of defenses by effectors can vary across plant species or varieties, i.e., some plant genotypes acquired the means to recognize particular effectors while others did not (Kant et al. 2015), and it was often suggested that this reflects the evolutionary arms races between plants and their attackers (Boller and He 2009). Also herbivores secrete mixtures of molecules that can simultaneously take effect as inducers and suppressors of plant defenses (Mattiacci et al. 1995; Halitschke et al. 2001; Hilker and Meiners 2006; Felton and Tumlinson 2008; Heil 2009; Schmelz et al. 2009; Consales et al. 2012; Maffei et al. 2012; Iida et al. 2019) yet extent to which such secretions result in a resistant or a susceptible plant differs across plant species and varieties. Several of such herbivore effector molecules have already been identified from their saliva or regurgitation fluids (Bos et al. 2010; Hogenhout and Bos 2011; Schmelz et al. 2012; Elzinga et al. 2014; Acevedo et al. 2015; Zhao et al. 2015; Villarroel et al. 2016; Chen et al. 2019; Su et al. 2019).

The ability of plants to activate defenses and to resist herbivores is not only constrained by their sensory arsenal but also challenged by all the remaining biotic and abiotic variations – stressful or not - in their habitat. One such variable is light. Light is the exclusive source of energy for carbon fixation via photosynthesis (Morker and Roberts 2011). Thereby it provides the energy needed for primary processes such as growth, development and reproduction and for secondary processes such as defenses (Griebel and Zeier 2008). Under light, a plant produces energy (ATP) and sugar using carbon dioxide via photosynthesis while, for most plants, this process largely reverses in the dark when energy is derived from burning the day-produced sugar using oxygen (and producing carbon dioxide) (Geiger and Servaites 1994). This major change coincides with large alterations in a substantial part of the plant’s remaining metabolism (Bläsing et al. 2005). In addition, plants also possess an internal circadian clock mechanism for the purpose of measuring time (Greenham and McClung 2015). The circadian clock has been investigated intensively; it drives diurnal cycles in a variety of ways independent from light or darkness and thus these will continue to cycle in constant light or darkness (Harmer 2009; Lu et al. 2017) until energy reserves run out.

Plant defense-responses induced by pathogens and herbivores are also subject to diurnal cycles (Karpinski et al. 2003; Downum 1992; Ballaré 2014) – either directly due to the absence/presence of light, to clock regulation or to both. In *Arabidopsis*, about 30% of the plant’s transcriptome exhibits a clock-regulated basal expression pattern and also hormonal pathways, such as the SA- and JA-signaling pathways and their downstream genes, appear to be under basic control of the clock. For example, in unwounded *Arabidopsis* plants, JA levels peaked at noon, while many JA-regulated genes, such as wound-responsive genes, were expressed highest at dusk. Alternatively, housekeeping SA levels in *Arabidopsis* peaked in the middle of the night (Covington et al. 2008; Goodspeed et al. 2012). In lima bean, it was shown that JA levels induced by artificial damage in leaves increased 2–3 times higher during the nocturnal phase than during the day (Arimura et al. 2008). Something similar was found for *Arabidopsis*, although the induction of the JA-marker gene *THI2.1* by avirulent *Pseudomonas syringae* pv. *maculicola* only occurred in the presence of light just as, interestingly, the induction of systemic acquired resistance by this pathogen (Zeier et al. 2004).

Less attention has been paid to the influence of light conditions on plant responses to herbivory. In some cases, resistance to herbivores appeared lower in darkness than under light. For example, chewing herbivory by the pine weevil *Hylobius abietis* was significantly larger on pine trees in the dark than under natural sunlight. Pines growing in sunlight produced more resin defenses in response to pine weevil feeding in the dark than those growing without light, and young pines were able to use stored carbon resources to accumulate chemical defenses in response to feeding damage (López-Goldar et al. 2016; Suárez-Vidal et al. 2017). The diurnal patterns of *Nicotiana attenuata* metabolites in response to larval feeding of the specialist herbivore *Manduca sexta* showed a tissue-specific pattern: root metabolites peaked during the night, whereas leaf metabolites peaked during the day (Kim et al. 2011). Furthermore, induced emissions of volatiles that attract natural enemies of herbivores were also found to be light dependent, with peak emission during the day and least emission in the night in some systems (Gouinguené and Turlings 2002; Loughrin et al. 1997; Maeda et al. 2000; Zhang et al. 2010). In addition, Goodspeed et al. (2012) observed that the larvae of *Trichoplusia ni* displayed circadian-controlled feeding behavior, peaking during the late day. A follow-up study showed that *T. ni* performs better on mutant plants lacking a circadian rhythm, suggesting the circadian clock enables plants to anticipate herbivore attack (Goodspeed et al. 2013a, b). Together these observations underpin that the absence of light at night as such can affect the plant’s ability to resist herbivores, whereas the plant’s clock mechanism can sometimes compensate for this by anticipating herbivore attack, for example herbivores with an activity peak around dusk or dawn.

In this study we compared key aspects of the mite-tomato interaction under light and in the dark. Experiments were prompted by the fact that practically all previous work on plant defenses in response to spider-mite feeding was performed by sampling mites and spider mite-infested plants half way during the light period of a 16/8-h day/night cycle (e.g., Glas et al. 2014; Kant et al. 2004; Liu et al. 2017). Using this sampling strategy we made generalized statements on defense induction and suppression by *T. urticae* and *T. evansi* (Alba et al. 2015; Godinho et al. 2016; Kant et al. 2008; Martel et al. 2015; Schimmel et al., 2018; Villarroel et al. 2016), on the expression of mite salivary effector-genes (Schimmel et al. 2017a), on mite feeding damage (Alba et al. 2015; Kant et al. 2004;) and on their rate of oviposition (Alba et al. 2015; Schimmel et al. 2017b) in relation to plant defenses. While the latter two parameters were always assessed across 24-h periods, the first two were only assessed during the middle of the light period. Therefore, alleged correlations between mite oviposition or plant damage and induction/suppression or effector production may have been estimated inaccurately. For example, spider mites are stylet feeders that were found to prefer to feed via stomata (Bensoussan et al. 2016) while the stomata of tomato, *Arabidopsis* and bean – all being C3 plants (Ehleringer and Monson 1993) - will close during the dark phase. In addition, *T. urticae* induces strong SA and JA responses during daytime whereas *T. evansi* suppresses these. However, since also these hormonal responses are under the influence of photoperiodism, this observation may not apply in the same way to the mite-plant interaction during the night. Moreover, we identified several secreted salivary effectors (Jonckheere et al. 2016, 2017). Two sets of secreted effector orthologs, i.e., Te28 and Te84 from *T. evansi* and Tu28 and Tu84 from *T. urticae*, were shown to account for suppression of JA and SA defenses in *N. benthamiana*, while their expression in spider mites was found to negatively correlate with the magnitude of induction in tomato (Villarroel et al. 2016; Schimmel et al. 2017a). However, also spider mites go through diurnal cycles and make use of photoperiodic time measurement (Veerman and Veenendaal 2003) causing their behavior (Ohtsuka and Osakabe 2009; Clotuche et al. 2011), oviposition (Polcik et al. 1965), physiology (Veerman 1993) and pesticide sensitivity (Fisher 1967) to vary across the day and the night (Vaz Nunes et al. 1990). For example, it was reported that spider mites initiate fewer feeding events during the dark than during the light period (Maeda et al. 2000). For reasons like these we decided to repeat our key experiments but sample not only in the middle of the light period but also in the middle of the dark period - at constant temperature and relative humidity - in order to compare plant defense-gene expression, mite effector-gene expression and mite feeding damage under two basal, but significantly different, conditions.

## Methods and Materials

### Plant Growth and Herbivore Rearing

Tomato (*Solanum lycopersicum* cv. Castlemart) and bean (*Phaseolus vulgaris* cv. Speedy) seeds were germinated and grown in the greenhouse [25°C, 16/8 h light/dark, 50–60% relative humidity (RH)]. After 10 days, tomato plants were transferred to climate rooms (23°C, 16/8 h light/dark, 60% RH, 300 µmol m^− 2^s^− 1^). For experiments, two groups of tomato plants were submitted to the same albeit reversed day-night cycle: room A from 6 pm to 10 am (16 h light) and from 10 am to 6 pm (8 h dark), which was used for sampling leaf material for assessing gene expression in darkness and for measuring leaf damage in dark phases and in light phases; and room B from 8 am to 12 pm (16 h light) and from 12 pm to 8 am (8 h dark), which was used for sampling leaf material and for assessing gene expression under light. Three-week-old plants were used for the experiments.

The *T. urticae* Koch spider mite line Santpoort-2 (Alba et al. 2015) was used as an inducer of defenses and the *T. evansi* Baker & Pritchard line Vicosa-1 (Alba et al. 2015; Sarmento et al. 2011a, b) was used as a suppressor of defenses. *T. urticae* Santpoort-2 was reared on detached leaves of *P. vulgaris* cv. Speedy while *T. evansi* Viçosa-1 was reared on detached leaves of *S. lycopersicum* cv. Castlemart. Cultures were placed on wet cotton wool and maintained in a climate room at 23 °C with a 16/8 h light/dark photoperiod, 60% RH and 300 µmol m^− 2^s^− 1^). The spider mites used for these experiments were derived from egg waves obtained by allowing adult females to produce eggs during 2 days. These egg waves were produced in the same climate room where they were used for the experiment to make sure they grew up under the same rhythmic light-dark conditions as the tomato plants.

### Characterization of Defense Gene Expression in the Light and Dark Phases

For performing RT-qPCR on tomato RNA, *actin* was used as reference gene, while the Cathepsin-D-inhibitor/chymotrypsin inhibitor encoding gene *Jasmonate-inducible Protein-21* (*JIP-21*) (Lisón et al. 2006), *Proteinase Inhibitor IIc* (*PI-IIc*) and *Proteinase Inhibitor IIf* (*PI-IIf*) (also called *WIPI-II*; Farmer et al. 1992) and SA-related *PR-1a* (Van Loon and Van Strien 1999) were used as defense marker genes. Primer sequences can be found in Alba et al. (2015). To evaluate defense gene expression in the light and dark phases and to sample them during daytime, plants were placed in two rooms with the same but reversed day-night cycles as explained in the previous section. Two-day-old adult female mites were produced as described in Alba et al. (2015) and transferred to the same room where the plants were to be used for the experiments. It took around 2 h to finish the infestation in each room (room B for light sampling: 10:00–12:00 h; room A for dark sampling and leaf damage assay: 12:00–14:00 h). For dark period samples, we took the plants out of the room and infested them in the climate room nearby which was not dark; after infestation we put the plants back in room A. Since we did this plant-by-plant each individual plant was exposed to light for only a few minutes. Three-week-old tomato plants were infested with 15 adult female spider mites per leaflet, three leaflets per plant, using five plants per treatment. The infested leaflets were isolated by a lanolin barrier at the petiole. At 4 days post-infestation, infested plants in both light and dark phases (i.e., 8 h after the beginning of the light treatment and 4 h after the beginning of the dark treatment) were sampled. Sampled leaflets were flash-frozen in liquid nitrogen and stored at -80 °C until mRNA was extracted. These experiments were repeated three times independently. qPCRs were performed on all samples separately and the data of three replicates were combined for gene expression analysis. All data were log transformed and tested for homogeneity of variances prior to data analysis. Differences in actin-normalized defense gene expression levels between light and dark phases of each mite strain were analyzed by Student’s t-test using IBM SPSS Statistics 22.

### Leaf Damage Assay

To assess the level of feeding damage inflicted by spider mites in dark and light phase, 21-day-old tomato leaflets were infested with 15 adult female spider mites (*T. urticae* or *T. evansi*) using five plants per treatment. We used only one of the two rooms for these observations, i.e., the room indicated as room A earlier. Infested leaflets were isolated by a lanolin barrier at the petiole. We then periodically photographed the leaves at the beginning and the end of the light phase in order to afterwards analyze the area covered by chlorotic lesions per hour during light and darkness. We infested the plants for the leaf damage assay between 12:00 and 14:00 h, and the first photos were taken after the plants had experienced the first dark period at the infestation day (grey bars in Fig. 3.2). We assumed that the mites were still habituating during this period, so we excluded this first time point from the analyses. Then, photos were taken every day during 4 days. Each photo included a 4-cm^2^ millimeter paper that served as a reference to later on convert pixels into mm^2^. Photos were analyzed using a similar protocol as in Kant et al. (2004) but we used a different software tool, ImageJ (Rasband 2012, http://rsb.info.nih.gov/ij/). For assessing the area of chlorotic damage, we first set the scales according to the reference to convert pixels to mm^2^. Then, all (colored) pixels were transformed to black-and-white using the threshold tool in such a way that all damaged areas were set to white and the remaining undamaged leaf area was set to black. The histogram tool was then used to count the white pixels (chlorotic damage) and the black pixels (intact leaf) of each separate leaflet. This experiment was repeated three times independently and data from three replicates were pooled for analysis.

The average amount of damage per hour (mm^2^ h^− 1^) was calculated for every consecutive light and dark period. We also analyzed the cumulative damage for the light and the dark period during a period of 4 days for both mite species. The cumulative mite-inflicted damage at end time points (t = 100 h) were tested by Student’s t-test. Linear regression was used to fit for the relationships of cumulative damage of dark and light phases over time respectively. To make the two regression lines clearer to compare, both light and dark series were set to start from the same time point. Slope test of the linear regression was used to assess the statistical relationships between dark and light series (Sokal and Rohlf 1995). Total average damage per hour was analyzed by using a Student’s t-test.

### Characterization of Spider Mite Effector Gene Expression in the Light and Dark Phases

We evaluated the expression levels of four candidate spider mite effector genes (*Tu28, Te28, Tu84, Te84*) (Villarroel et al., 2016) during the light and dark phases by means of RT-qPCR. For both spider mite species, the large subunit ribosomal protein 49 (*rp49*) was used as the housekeeping gene. All primers had been tested for mite specificity. Primer sequences can be found in Table S3 of Schimmel et al. (2017). We used RNA from the same mite-infested leaflets that were analyzed for defense gene expression (since these are mixtures of mite and tomato RNA). Differences of means between different light and dark phases of each mite strain were analyzed by Student’s t-test.

## Results

### In darkness relative to light, expression levels of defense genes were lower in *T. urticae* infested plants whereas the expression level of *PR1a* was higher in *T. evansi* infested plants 

**Fig. 1:**
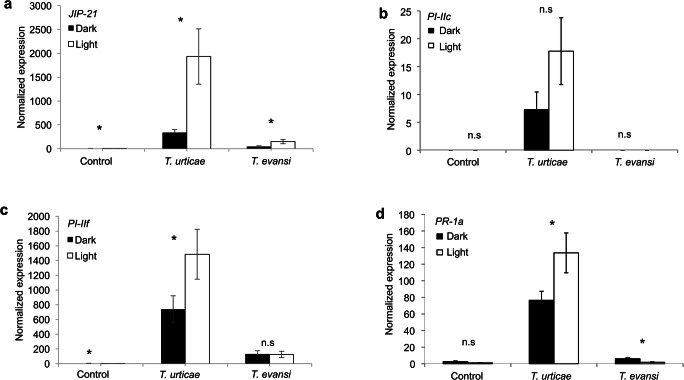
Expression of defense-related genes in *T. evansi-* and *T. urticae-* infested tomato leaflets under light or darkness after 4 days of infestation. Panels show JIP-21 (**a**), *PI-IIc* (**b**), *PI-IIf* (**c**) and *PR-1a* (**d**) transcript levels normalized to actin. Uninfested leaflets were used as controls. Infested leaflets were sampled in the middle of the dark or light phase. Bars represent the means (± SE). All bars were scaled to the lowest average (thereby setting the lowest to 1). Asterisks represent significant differences between expression levels under light and darkness according to Student’s *t*-test (*P*≤ 0.05), n = 15, n.s = not significant

*T. urticae* induced significantly higher expression of JA-marker genes *JIP-21* (Fig. 1a; *t*-test: *t*_*27*_ = -3.918, *P* < 0.001) and *PI-IIf* (Fig. 1c; *t*-test: *t*_*27*_ = -2.012, *P* = 0.027) in light than in dark. Although this trend was sustained for the gene *PI-IIc*, it was not significant (Fig. 1b; *t*-test: *t*_*25*_ = -1.7, *P* = 0.102). The same trend was found for the SA-marker gene *PR-1a* (Fig. 1d; *t*-test: *t*_*28*_ = -2.164, *P* = 0.039). In addition, *T. evansi* upregulated the SA-marker gene *PR-1a* to a lower level in light than in the dark (Fig. 1d; *t*-test: *t*_*28*_ = 3.674, *P* = 0.001). In contrast, expression levels of the JA-marker gene *JIP-21* in plants infested with *T. evansi* were lower in the dark than in the light (Fig. 1a; *t*-test: *t*_*28*_ = -2.769, *P =* 0.005).

### *T. evansi* Damaged Tomato More Heavily than *T. urticae* and Both Mites Caused More Chlorotic Feeding Damage in Darkness than in Light

**Fig. 2:**
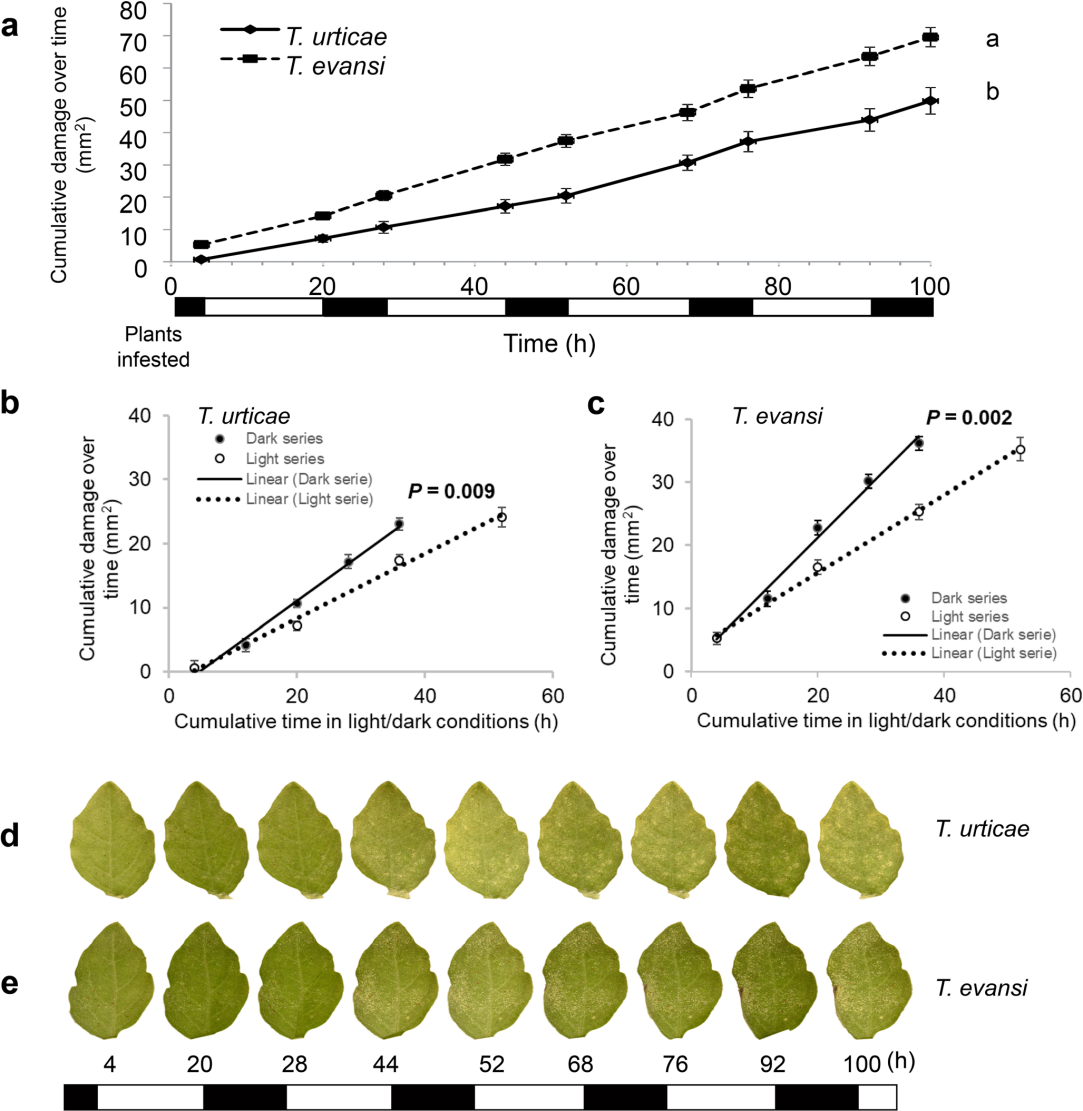
Cumulative damage by *T. urticae* and *T. evansi* feeding on tomato leaflets over a period of 100 h**.** Leaf area damaged by spider mites was determined from 15 plants infested with 15 adult females of *T. urticae* (**a**, solid line) and *T. evansi* (**a**, dotted line). Feeding damage was measured at the beginning and the end of each light phase. Shown are the damaged leaflets at each measuring time point (**d, e**). Curves with different letters differ significantly at end time point according to Student’s *t*-test (*P* < 0.05), n = 15 (**a**). Vertical bars represent SE (**a**). Average damage increment per time point was calculated, shown are the cumulative damage between samples in cumulative time points, the damage in dark (**b, c**, solid circles) and light (**b, c**, empty circles) phases were separated and linear regression was used to test whether regression slopes of dark (**b, c**, solid line) and light series (**b, c**, dotted line) were statistically different by regression analysis (Sokal and Rohlf 1995). Error bars represent the standard error (SE) observed in each time point

 The cumulative damage inflicted by *T. urticae* and *T. evansi* over 4 days after infestation is shown in Fig. 2. The specific time when photos of the amount of feeding damage were taken are indicated in Fig. 2d and e. *T. evansi* caused significantly more damage than *T. urticae* at the end of 4 days of feeding (t = 100 h) (Fig. 2a; *t-*test: *t*_*28*_ = -3.875, *P* = 0.001), a result that was also found in Alba et al. (2015) who showed that *T. evansi* inflicted more than twice the amount of feeding damage as *T. urticae*. Interestingly, *T. urticae* caused more damage in dark than in light (Fig. 2b; regression slope analysis: *Fs* = 17.53, *P* = 0.009), the same trend was found for *T. evansi* (Fig. 2c; regression slope analysis: *Fs* = 38.45, *P* = 0.002).

### Total Average Damage Caused by Spider Mites per Hour was Higher in Darkness than in Light

**Fig. 3:**
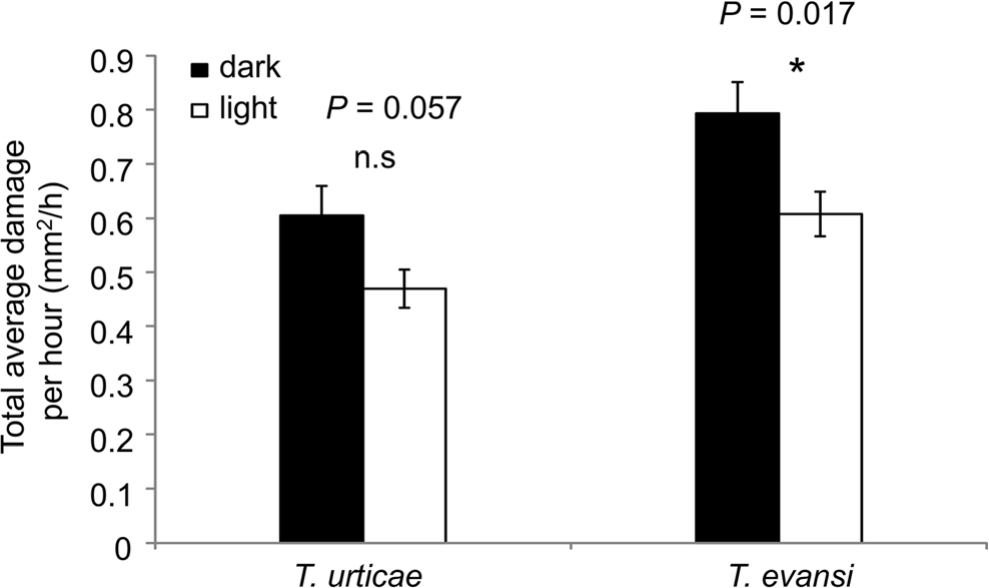
Average cumulative damage per hour on tomato leaflets infested with *T. urticae* or *T. evansi*. Leaf area damaged by spider mites was determined from 15 plants infested with 15 adult females of *T. urticae* (**a**) and *T. evansi* (**b**). Feeding damage was measured at the beginning and the end of each light phase. Bars represent the average damage increase per hour (± SE) of all sampling dark/light phase. Bars capped with * were significantly different according to Student’s *t*-test (*P*≤ 0.05), n = 60. n.s = not significant

 The average damage per hour of all the light or dark period samples over the course of the experiment is shown in Fig. 3. *T. evansi* caused significantly more feeding damage per hour under dark than under light conditions across the experiment (Fig. 3; *t*-test: *t*_*118*_ = 2.421, *P* = 0.017). Feeding damage by *T. urticae* showed a similar pattern although this result is only marginally significant (Fig. 3; *t*-test: *t*_*118*_ = 1.92, *P* = 0.057).

### Expression Levels of Spider Mite Effector Genes *Tu84* and *Te84* Were Higher in Light than in Darkness

**Fig. 4:**
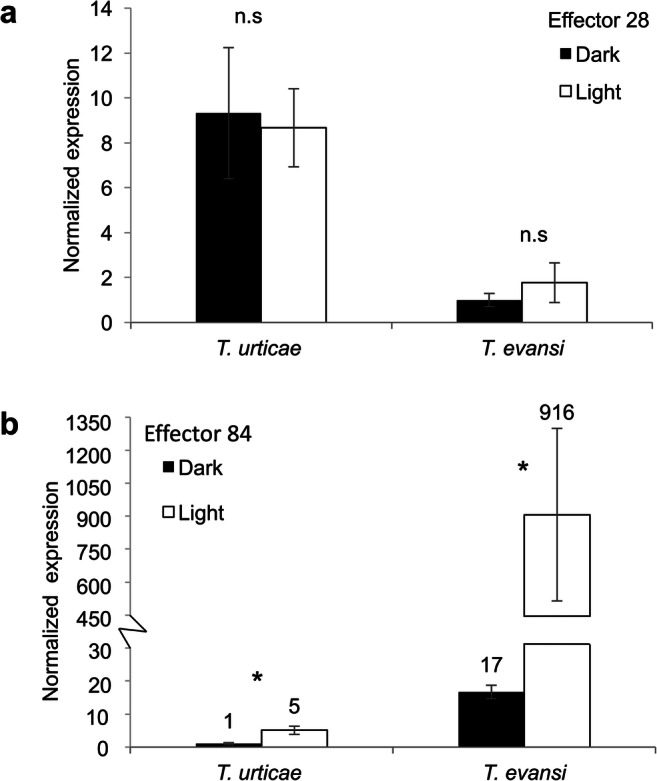
Expression of spider mites effector genes in *T. evansi* and *T. urticae* when feeding from tomato leaflets during the light or the dark phase. *Tu28* and *Te28* (**a**), *Tu84* and *Te84* (**b**) transcript levels were normalized to ribosomal protein 49. Infested leaflets with 15 adult female spider mites were sampled in the middle of the dark or the light phase. Bars represent means (± SE) and all bars were scaled to the lowest average (thereby setting the lowest to 1). Asterisks represent significant differences between expression levels under light and darkness according to Student’s *t*-test (*P*≤ 0.05), n = 15, n.s = not significant

 There were no significant differences between *Tu28* and *Te28* expression levels in light and dark (Fig. 4a; *t*-test: *P* > 0.05). Transcript accumulation of effector gene *Tu84* was higher in light than in dark (Fig. 4b; *t*-test: *t*_*8*_ = -3.544, *P* = 0.008), and the same applied to *Te84* (Fig. 4b; *t*-test: *t*_*8*_ = -2.957, *P* = 0.018). In addition, the expression level of *Te84* was also much higher than that of *Tu84* (Fig. 4b)..

## Discussion

Here we have shown that both induction and suppression of the defense of tomato plants by spider mites, as observed under light, are also retained in the dark, albeit at a lower absolute magnitude. This finding is consistent with the observation that the amount of leaf area covered with chlorotic lesions produced per hour by feeding spider mites increased faster in dark, when defenses are lower, than in light. We further observed that expression levels of the mite effector gene *84*, but not effector gene 28, were significantly higher under light than under darkness for both *T. urticae* and *T. evansi.*

We used two species of spider mites, viz., *T. urticae* Santpoort-2, which poorly performs on tomato, and *T. evansi* Vicosa-1, which thrives on tomato. Our results showed that *T. urticae* induces the expression of the JA-marker gene *JIP-21, PI-IIc, PI-II*f, and the SA-marker gene *PR-1a* strongly during the light phase when compared to the dark phase. In the uninfested control plants, expression of *JIP-21* and *PI-IIf* was also higher in the light than in the dark albeit at very low absolute levels (Fig. 1a and c). Previously, we showed that this mite strain has better performance in the absence of these defenses (Kant et al. 2008; Alba et al. 2015). This suggests that darkness may provide the mites with a relative benefit, supported by an overall higher degree of feeding damage in darkness (Figs. 2 and 3). Similarly, suppression of *PR-1a* by *T. evansi* was stronger during the light phase than during the dark phase and this also coincided with a higher level of feeding damage under darkness. These results contradict experiments of Maeda et al. (2000) who scored the number of feeding events by *T. urticae* in light and in darkness by monitoring the movement of fluid in their bodies and concluded that they feed more under light conditions. However, Bensoussan et al. (2016) claim that the frequency of feeding events involving multiple cells correlates negatively with the number of dead cells. This would imply that a higher number of feeding events does not necessarily indicate more feeding. Furthermore, Bensoussan et al. (2016) suggest that the white feeding-damage spots are largely due to an indirect effect of feeding: these chlorotic cells merely surround the emptied cell but were not used by the mite to feed on. Although we feel it is safe to assume that the amount of chlorotic lesions will correlate positively with the number of cells that were emptied for food (see also Park and Lee 2002), the observations of Bensoussan et al. (2016) do suggest that this phenotype may not reflect feeding damage as such but more a response of the plant to feeding damage.. If so, this response could possibly be stronger in darkness. We do not know the physiological cause of this spot formation around emptied cells but it reminds of responses related to senescence/apoptosis (see Alba et al. 2015 for a description). Senescence in intact tomato plants, and in detached *Arabidopsis* leaves, can be induced by various treatments, including those with the hormones ethylene, JA, and SA, but also darkness can induce senescence (Lira et al. 2017; Weaver and Amasino 2001). Therefore, despite the clear positive correlation between these chlorotic lesions and mite performance (Kant et al. 2004; Alba et al. 2015), we cannot exclude that lesion formation is amplified by darkness. Spider mite feeding correlates with their rate of oviposition and it has been observed that spider mite oviposition decreases in the dark (Polcik et al. 1965; Maeda et al. 2000). In theory we could test if oviposition, as a proxy of feeding activity, correlates with lesion formation. However, we –feel this would not be informative as plant material can take several days to be digested (Storms 1971) and any diurnal pattern in egg production can therefore not be linked directly to the quality of the plant material eaten during the previous hours. This would require, among other things, for example experiments where leaflets are infiltrated with radio-labeled amino acids to measure the label back in mites at the end of a light or dark cycle to get a more complete picture of the actual feeding activities of the mites. Yet, it would be difficult to disentangle plant quality from a direct effect of light/darkness on the feeding behavior of the mite since also mites display circadian rhythms (Veerman and Veenendaal 2003) and thus would require using mites displaying a reversed diurnal cycle as well. Overall, our results indicate that plant defense responses to spider mites vary diurnally although these variations do not alter the observation that *T. urticae* generally induces, and *T. evansi* generally suppresses, key tomato defenses (Alba et al. 2014, Schimmel et al. 2018). It is feasible, however, that a denser time series would reveal larger differences. For example, in *Arabidopsis* the accumulation of JA and the expression levels of many JA-regulated genes peak at dawn (Shin et al. 2012). This is interesting because it was also observed that spider mites display a strong diurnal gradient of sensitivity to pesticides and this sensitivity is high at dawn (Fisher 1967; Polcik et al. 1964). The feeding-activity pattern of both spider mite species aligns with previous findings in other systems. Many invertebrate herbivores are more active during the night than during the day. For example, *Arabidopsis* plants grown in the field were attacked by slugs predominantly during the night (Baldwin and Meldau 2013) and pine trees were found to be attacked more frequently by the pine weevil *Hylobius abietis* in darkness than under natural sunlight (Lopez-Goldar et al. 2016). It has been proposed that such behaviors could serve to avoid predators or parasitoids that strongly depend on vision (Hassell and Southwood 1978). In addition, it was found that crops grown in dense populations promote herbivore growth and development. This was attributed to the effect of shade-mediated competition where plants may prioritize outgrowing competitors over defense (Moreno et al. 2009; Roberts and Paul 2006).

Already during the first hours of our experiment *T. evansi* had inflicted more feeding damage than *T. urticae* (Fig. 2a) most likely because the first is adapted to tomato while the latter is not (Alba et al., 2015). Spider mites were shown to frequently reach the mesophyll cells by inserting their stylets into the open stomata (Bensoussan et al. 2016). It has been suggested that the fact that C3 plants close their stomata during the night automatically creates a pre-invasive physical barrier that restricts pathogens to invade via the stomata and thus may allow a plant to relax its defenses during the night (Zhang et al. 2013). Thus, our observation that the magnitude of induced defenses decreases at night could also reflect such a relaxation response of the plant after it closed its stomata. However, the increase in lesion-size formation and the fact that mites just as well can reach the mesophyll via the epidermis, carefully avoiding to damage epidermal cells (Bensoussan et al. 2016), argues against the suggestion of Zhang et al. (2013) also to apply to spider mites. It is also possible that at night not only the mite’s ability to reach the appropriate tissues and the plant’s ability to handle this stress efficiently but also the nutritional quality of the plant as a host are affected. Spider mites need amino acids, sugar (sucrose), vitamins, salts and lipids (sterols) in their diet (Bosse 2010). Unfortunately, there is too little detailed information on these dietary requirements to speculate on how a plant’s diurnal cycle could affect its quality as a mite diet and it would be challenging to couple the diurnal supply of nutrients in the plant to the diurnal nutritional intake of the herbivore not knowing which nutrients matter to the mite and which do not. Nevertheless, diurnal fluctuations in diet quality rather than defenses may impact spider mite’s feeding activity as well but identifying key metabolites may require detailed temporal analyses of the metabolomes – and the interactions therein - of both plant and mite in relation to fitness parameters such as feeding damage or reproductive performance.

We also observed light dependent variation in expression levels of the mite effector genes *Tu84* and *Te84*, with especially the *T. evansi* ortholog being expressed much lower in darkness. We did not come across studies that explicitly examined the diurnal expression of effector genes of herbivores or plant pathogens. Yet, pathogenicity of plants often appeared to be light-dependent (Bonomi et al. 2016; Río-Álvarez et al. 2014; McClung 2011), and it has been known for decades that plant microbial pathogens display diurnal rhythms of infection activity (Martinez-Bakker and Helm 2015; Sreeramulu 1959) while the intact circadian clock was found to be essential for virulence of *Botrytis cinerea* (Hevia et al. 2015). Gene *Te84* has been previously shown to be upregulated in *T. evansi* upon introduction of *T. urticae* on the same tomato leaflet and this resulted in enhanced local suppression of defenses near the feeding site of *T. evansi* (Schimmel et al. 2017a, b). Clearly, the expression of *Te84* is highly plastic which is reminiscent of the salivary SHOT (Secreted Host-responsive protein Of Tetranychidae) genes of the mite (Jonckheere et al. 2018). Whether there is a causal relationship between the decreased expression of salivary effector genes *Tu84* and *Te84* during the night and the increased feeding damage remains speculative. Moreover, it is currently not known whether these saliva effector proteins have other functions in mites in addition to the effect they have on plant defenses. However, it is clear that for understanding the relationship between rhythmic and incidental transcriptional plasticity of effector genes and the induced defense-responses in host plants that go through diurnal cycles, it will be necessary to not only rely on correlation studies but also on the ability to functionally knock-out or knock-down the genes (traits) that play key roles.

Comparing biotic interactions under light and in darkness is not the same as comparing them during the day and the night. At night not only the light intensity but also, for example, the amount of UV, the temperature and humidity differ from those during the day. Spider mite development slows down at lower temperatures (Margolies and Wrensch, 1996) and also the amount of feeding damage they cause correlates positively with temperature (Candolfi et al. 1991). Also plant defenses can be profoundly affected by temperature (Colhoun 1973) although tomato JA-defenses were suggested to be fairly robust when attacked by caterpillars under different temperatures (Havko et al. 2020). Therefore, in nature the effects of darkness we observed in our study can be modulated in different ways depending on the different (abiotic) characteristics of local environments.

In summary, in many plant-pathogen and plant-herbivore studies it was shown that both light and circadian clock contribute to the outcome of their interaction. Herbivory may be restricted by diurnal fluctuations in a plant’s nutritional quality and by diurnal variation in resistance while, in contrast, they may also take advantage of these diurnal programs by attacking a plant, or by increasing their feeding activities, when it is most vulnerable. Our results showed that effector genes *Tu84* and *Te84* are expressed much higher in light than in dark while both mite species caused overall more damage during the dark phase. Hence, we speculate that these mites may relax effector production during the dark phase because under these conditions the plant’s ability to upregulate defenses is reduced. Results suggest that SA- and JA-regulated responses are subject to diurnal variation possibly causing tomatoes to be more susceptible to spider mite feeding during the dark phase.
